# Advanced Imaging for Quantitative Evaluation of Aphanomyces Root Rot Resistance in Lentil

**DOI:** 10.3389/fpls.2019.00383

**Published:** 2019-04-16

**Authors:** Afef Marzougui, Yu Ma, Chongyuan Zhang, Rebecca J. McGee, Clarice J. Coyne, Dorrie Main, Sindhuja Sankaran

**Affiliations:** ^1^Department of Biological Systems Engineering, Washington State University, Pullman, WA, United States; ^2^Department of Horticulture, Washington State University, Pullman, WA, United States; ^3^United States Department of Agriculture-Agricultural Research Service, Grain Legume Genetics and Physiology Research Unit, Washington State University, Pullman, WA, United States; ^4^United States Department of Agriculture-Agricultural Research Service, Plant Germplasm Introduction and Testing Unit, Washington State University, Pullman, WA, United States

**Keywords:** disease severity, RGB imaging, hyperspectral imaging, multispectral imaging, remote sensing, feature selection

## Abstract

Aphanomyces root rot (ARR) is a soil-borne disease that results in severe yield losses in lentil. The development of resistant cultivars is one of the key strategies to control this pathogen. However, the evaluation of disease severity is limited to visual scores that can be subjective. This study utilized image-based phenotyping approaches to evaluate *Aphanomyces euteiches* resistance in lentil genotypes in greenhouse (351 genotypes from lentil single plant/LSP derived collection and 191 genotypes from recombinant inbred lines/RIL using digital Red-Green-Blue/RGB and hyperspectral imaging) and field (173 RIL genotypes using unmanned aerial system-based multispectral imaging) conditions. Moderate to strong correlations were observed between RGB, multispectral, and hyperspectral derived features extracted from lentil shoots/roots and visual scores. In general, root features extracted from RGB imaging were found to be strongly associated with disease severity. With only three root traits, elastic net regression model was able to predict disease severity across and within multiple datasets (*R*^2^ = 0.45–0.73 and RMSE = 0.66–1.00). The selected features could represent visual disease scores. Moreover, we developed twelve normalized difference spectral indices (NDSIs) that were significantly correlated with disease scores: two NDSIs for lentil shoot section – computed from wavelengths of 1170, 1160, 1270, and 1280 nm (0.12 ≤ |*r*| ≤ 0.24, *P* < 0.05) and ten NDSIs for lentil root sections – computed from wavelengths in the range of 630–670, 700–840, and 1320–1530 nm (0.10 ≤ |*r*| ≤ 0.50, *P* < 0.05). Root-derived NDSIs were more accurate in predicting disease scores with an *R*^2^ of 0.54 (RMSE = 0.86), especially when the model was trained and tested on LSP accessions, compared to *R*^2^ of 0.25 (RMSE = 1.64) when LSP and RIL genotypes were used as train and test datasets, respectively. Importantly, NDSIs – computed from wavelengths of 700, 710, 730, and 790 nm – had strong positive correlations with disease scores (0.35 ≤*r* ≤ 0.50, *P* < 0.0001), which was confirmed in field phenotyping with similar correlations using vegetation index with red edge wavelength (normalized difference red edge, 0.36 ≤ |*r*| ≤ 0.57, *P* < 0.0001). The adopted image-based phenotyping approaches can help plant breeders to objectively quantify ARR resistance and reduce the subjectivity in selecting potential genotypes.

## Introduction

Lentil (*Lens culinaris* Medik.) is a leguminous crop grown worldwide that serves as an important source of protein for human consumption and animal feed (Infantino et al., 2006; Hamwieh et al., 2009). The overall annual production worldwide reached approximately 6 million tons in 2016 with 3 million tons produced in Canada alone (Food and Agriculture Organization of the United Nations [FAO], 2016). However, as with other crop species, lentil is subject to numerous abiotic and biotic stresses. In breeding, crop performances, including stress tolerance/resistance, are screened based on well-developed root systems (e.g., root length, number of lateral roots), wilting score (Idrissi et al., 2016), early vigor, yield potential (Tullu et al., 2001; Rodda et al., 2017), seed shape, seed weight (Subedi et al., 2018), and disease resistance (Rubeena et al., 2006; Tivoli et al., 2006). Soil-borne fungal diseases are one of the limiting factors negatively affecting plant development and seed yield in pulses (Gossen et al., 2016). These pathogens can attack their host at any stage causing great loss in yield (Infantino et al., 2006). The severity of some root rot diseases may not be visible at early growth stages, as the initial infection occurs in roots and the expression of above ground symptoms may be delayed (Gossen et al., 2016; Bodah, 2017). Among these diseases, Aphanomyces root rot (ARR), caused by *Aphanomyces euteiches* Drechs., is one of the most serious diseases affecting legume production (Gossen et al., 2016). The damage can lead up to 100% yield loss in pea production (Gaulin et al., 2007). This oomycete causes rotting of roots and epicotyls, which results in stunted plant growth, yellow leaves, and reduced pod fill and production (Ford et al., 1999; Pilet-Nayel et al., 2002; Gaulin et al., 2007; Gossen et al., 2016; Bodah, 2017). The severity of *A. euteiches* is highly influenced by environmental conditions and agronomic practices. Soil moisture increases the incidence of disease proliferation (Ford et al., 1999) and chemical controls alone are not enough for managing the disease (Pilet-Nayel et al., 2002; Wu et al., 2018). Additionally, the current high yielding lentil lines show little resistance to *A. euteiches* (Moussart et al., 2008; Vandemark and Porter, 2010). The domestication of lentils has also led to a loss in genetic diversity, including the loss of some important traits contributing to disease resistance (Ford et al., 1999; Khazaei et al., 2016). For these reasons, the development of disease-resistant cultivars is a critical need for crop protection against this pathogen (Ford et al., 1999; Infantino et al., 2006; Le May et al., 2017).

Different screening methods have been used to study the phenotypic characterization of large sets of genotypes and to select the desirable traits (Furbank and Tester, 2011). The current assessment relies on visual estimates of disease severity and effects on the whole plant (Pilet-Nayel et al., 2002; Bani et al., 2012). However, given that large numbers of plants need to be evaluated, the conventional phenotyping process – using resistance scoring or direct measurements – is laborious, time-consuming (Adu et al., 2014), and can be subjective depending on the expertise of the plant breeder. A different approach, such as high-throughput phenotyping using imaging techniques for objective and quantitative selection of disease resistance, offers new opportunities to change the subjective evaluation and to discern minute differences in plant responses that visual assessments are unable to capture.

Various sensing and imaging techniques were studied for stress detection, in both controlled environment and field conditions (Li et al., 2014; Sankaran et al., 2015). The rapidly evolving technologies, including digital Red-Green-Blue (RGB), fluorescence, multi/hyperspectral, and thermal imaging (Sankaran et al., 2010; Mahlein, 2016) coupled with advances in pattern recognition and machine-learning approaches (Singh et al., 2016) have increased the potential to phenotype thousands of plants in a high-throughput manner. For example, Naik et al. (2017) used RGB imaging to extract yellow and brown color percentages from soybean leaves to distinguish between severity classes of iron deficiency chlorosis. The described research tested the performance of 10 different machine-learning classifiers on a set of 4500 images and found that hierarchical models were able to generate a mean per class accuracy of 95.5%. RGB imaging techniques were also used to extract morphological traits and study root architecture. Desgroux et al. (2018) employed root system architecture (RSA) traits extracted from RGB images in a genome-wide association mapping population to identify quantitative trait loci (QTL) responsible for resistance to *A. euteiches* in pea plants. A color scanner was used to scan roots at 300 dpi and images were analyzed with Winrhizo^^®^^ software (Regent Instruments Inc., Quebec, Canada) to extract four main traits: total root projected area, total root length, average root diameter, and average lateral root length. They found that RSA traits were negatively correlated with Aphanomyces susceptibility and discovered one highly significant single-nucleotide polymorphism (SNP) marker associated with both root rot resistance and total root projected area. Shovelomics (Trachsel et al., 2011) integrated with RGB imaging (Bucksch et al., 2014; Das et al., 2015; Burridge et al., 2016; Saengwilai et al., 2018) have been used to extract root features under field conditions.

Hyperspectral imaging, in contrast, covers a broader spectrum, including visible and near-infrared regions. Several studies have demonstrated the efficiency of hyperspectral techniques in predicting specific pigments, such as chlorophyll and carotenoid. For example, the pigment content extracted from the hyperspectral data was related to angular leaf spot disease in cucumber leaves with correlation coefficients (*r*) of 0.87 (Zhao et al., 2016a). Similarly, this imaging technique has been widely used for detecting diseases in plants (Baranowski et al., 2015; Calderón et al., 2015; Zhao et al., 2016b). However, studies on the applications of hyperspectral imaging for root phenotyping are limited. On the other hand, multiple studies have shown the potential of proximal remote sensing in evaluating disease resistance in field conditions including; using hyperspectral sensing for *Xylella fastidiosa* infestation in olives (Zarco-Tejada et al., 2018), multispectral and hyperspectral sensing for citrus greening disease detection (Kumar et al., 2012), and RGB and multispectral imaging to assess potato late blight resistance (Sugiura et al., 2016; Duarte-Carvajalino et al., 2018).

In this study, we investigated the feasibility of applying high-throughput phenotyping imaging techniques for an objective assessment of ARR severity symptoms in lentils for rapid and accurate identification of resistant genotypes. The objectives of this study were to: (1) evaluate the relationship between disease visual scores and digital features extracted from RGB imaging and hyperspectral imaging (550–1700 nm) using two independent panels of lentil genotypes in greenhouse (controlled environment) conditions; (2) develop models for selecting most relevant features for ARR severity; and (3) investigate the performance of unmanned aerial system (UAS)-based multispectral imaging in field conditions for ARR detection.

## Materials and Methods

### Plant Materials, Inoculation, and Ground Reference Data

In greenhouse conditions, RGB and hyperspectral sensing systems were evaluated using two independent experiments. The first experiment was conducted in 2017 (termed as GH_LSP), where a total of five replicates from a genome-wide association mapping panel of 351 lentil accessions (USDA lentil single plant-derived collection/LSP) were grown. Two treatments – control and inoculated with *A. euteiches* – were performed using a split-plot design, where genotypes were allocated to subplots. In the second experiment (termed as GH_RIL), a recombinant inbred line (RIL) population of 191 genotypes of lentils were grown using a completely randomized design in 2018. To increase the number of plants during evaluation, three replicates with three seedlings each were used in each treatment (control and inoculated). Both experiments were conducted in a greenhouse at Washington State University, Pullman, WA, United States. Temperatures were maintained at 25°C (day) and 18°C (night) with a photoperiod of 16 h.

**FIGURE 1:**
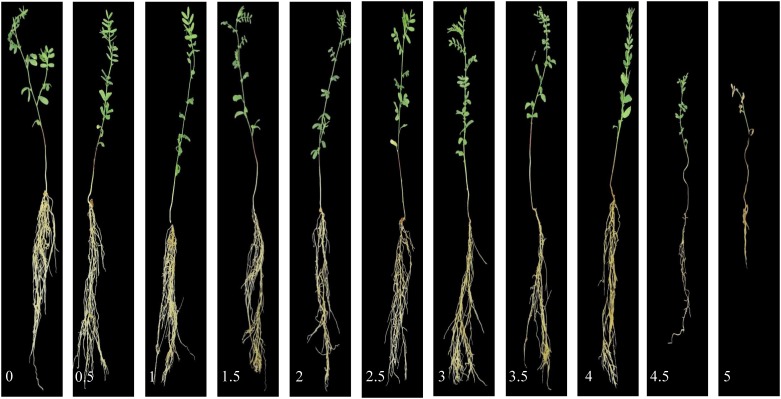
Aphanomyces root rot disease severity scale. Images were processed with background removed for better visualization.

Both lentil panels were used to evaluate resistance to a pure-culture strain of *A. euteiches* Drechs. F. sp. *pisi*, acquired from the USDA-ARS Grain Legume Genetics and Physiology Research Unit, Pullman, WA, United States. Zoospores were produced following the method described by Wicker et al. (2001). Zoospore concentration was adjusted to 1 × 10^4^ spores per mL. Prior to planting, the seeds were surface disinfected with 95% ethanol for 1 min, 10% sodium hypochlorite for 1 min, and rinsed thoroughly with distilled water. The seeds were planted in containers filled with perlite. The 14-day-old seedlings were inoculated with the zoospore suspension by pipetting 2 mL of inoculum, while non-inoculated controls were inoculated with 2 mL of sterile distilled water. The two groups of treatments (control and inoculated) were kept on different benches in the same greenhouse chamber. Fourteen days after inoculation, the plants were removed from the containers, the perlite washed off, and the roots scored using 0–5 disease scoring scale (standard phenotyping protocol) adapted from McGee et al. (2012). A score of 0 indicates no visible symptoms and a white root; 0.5 indicates less than 5% of discolored lesions on the entire root system; 1 indicates 5–15% of discolored lesions on the entire root; 1.5 indicates 15–25% of discolored lesions on the entire root; 2 indicates 25–50% minor discoloration on entire root system; 2.5 indicates 50–75% major discoloration on entire root system; 3 indicates more than 75% of brown discoloration on entire root system; 3.5 indicates brown discoloration on entire root system with some symptoms on hypocotyl; 4 indicates brown discoloration on entire root system with a shriveled and brown hypocotyl; 4.5 indicates brown discoloration on entire root system with a shriveled, brown, and soft hypocotyl; and 5 indicates a dead plant (Figure 1 and Supplementary Figure S1). After visual scoring and imaging, shoots and roots (combined by each replicate) were separated and kept in a drying room for 1 week at 60°C before collecting dry weight data (shoot dry weight/SDW, root dry weight/RDW).

The field evaluation was conducted in 2018 (termed as FE_RIL) using genotypes grown in a grower’s naturally infected field located near Kendrick, Idaho (46° 35′ 37.26″N, 116° 33′ 42.26″W). The genotypes were the 173 RILs (evaluated in greenhouse GH_RIL as noted from previous section). It was planted on 13 May 2018, with two parental lines and a variety check (Avondale), using a randomized complete block design with three replicates and 30 seeds per replicate. Plots consisted of six 1.5 m long rows with 19 cm row spacing. Within each plot, the rows 1 and 6 were the check cv “Avondale” and rows 2–4 were the RILs. Every 10th plot, winter wheat replaced Avondale in row 6. Avondale was used as reference to account for the variations in field when calculating the adjusted visual score, while the winter wheat was used as a marker for orientation and to separate replicates (two plots of wheat). Visual rating of lentils for resistance to ARR was based on above ground index (AGI). The AGI was evaluated for each row using a 1–5 scale adopted from Pilet-Nayel et al. (2002) with 1 as healthy; 2 as slight yellowing of lower leaves; 3 as necrosis of the lower leaves, some stunting, a few dead plants; 4 as necrosis of at least half or more of the plants with stunting, more than half of the row dead; and 5 as all plants dead or nearly so. The visual scores were utilized as ground reference data in both greenhouse and field conditions.

### Greenhouse Experiments: RGB Imaging, Image Processing, and Feature Extraction

A 16-megapixel digital camera (Canon^^®^^ PowerShot SX530 HS, a CMOS camera with maximum resolution of 4608 × 3456 pixels) was used for capturing high-resolution images at a distance of 508 mm from the plants. The camera was mounted on a metal frame to maintain stability. For uniform lighting conditions, a phenotyping box was used that was covered internally with white paper and illuminated by two fluorescent white lights (visible range 400–700 nm). The light sources were placed at the same level as the camera lens (Supplementary Figure S2). The setup was evaluated for image quality by comparing the area of roots extracted from digital images with plant biomass (dry weight) data and visual scores. The system produced a stable condition for automated image processing. During image collection, a white reference panel (Spectralon Diffuse Reflectance Standard, SRT-99-050, Labsphere, North Sutton, NH, United States) was also used for radiometric calibration to correct for variations in light intensity, if needed (∼99.9% reflectance). White balance was set to auto-mode throughout data collection. The image resolution was set to 0.17 mm/pixel. Up to five plants for GH_LSP experiment and six plants for GH_RIL experiment were captured in a single image frame. Image capturing was performed together with root uprooting and visual scoring.

**FIGURE 2:**
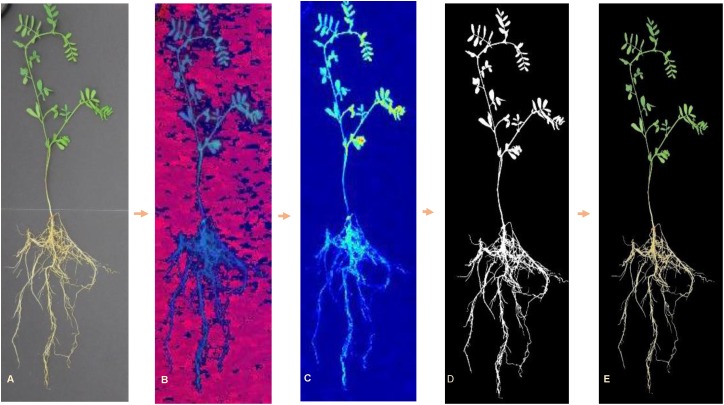
RGB image processing steps for background removal: **(A)** original RGB image, **(B)** HSV image, **(C)** hue channel image, **(D)** resulting mask, and **(E)** RGB image with background elimination.

All images were processed using customized algorithms written in MATLAB^^®^^ (2017a, The MathWorks, Natick, 2017), which enabled automated feature extraction. Images were, first, radiometrically corrected according to the values of reflectance panel and, then, converted from RGB color space to hue–saturation–value (HSV) color space for background segmentation. A color mask was created by combining a dual threshold from both hue and value channels. Pixels with green and yellow color were included into the plant mask (Hue < 0.45 and Value < 0.49) (Figure 2). Area filter was applied to remove small noises (pixels < 200); while keeping larger objects. Individual plants were cropped from each image and saved for feature extraction. This process resulted in 6299 images of individual plants (Table 1), since some of the seedlings did not emerge or develop.

**Table 1:** Summary of data analyzed in this study.

Imaging technique	Raw data size (GB)		USDA lentil single plant-derived collection	Recombinant inbred lines population
RGB (GH_LSP /GH_RIL)	5	Number of genotypes	351	191
		Number of images	3474	2825
Hyperspectral (GH_LSP /GH_RIL)	171	Number of genotypes	79	21
		Number of hypercubes	1379	705
Multispectral (FE_RIL)	3	Number of genotypes	–	173
		Number of images	–	1125

A total of 82 features were extracted from each image that included color, texture, and geometric features. For color features, all the background was set to NaN (not a number), and mean of pixel intensity, standard deviation, variance, and entropy of each channel were extracted from three different color spaces (RGB, HSV, and Lab, 36 features). In addition, these statistical descriptors were, also, extracted from grayscale images that were converted from RGB images (four features). For quantifying discoloration, the HSV color space was quantized into 7 hues × 2 saturations × 1 value, resulting in 14 different color bins or color combinations. The transformation into different bins reduces the number of used colors to represent an image, which helps in finding low-level color descriptors for the image (Zeng, 2016). Segment partitioning was conducted by selecting color intervals from the hue wheel. Only colors in the range of orange to green were selected, which corresponded to hue in the interval [0.05, 0.40]. The seven intervals were determined based on the distribution of color pixels in Hue channel from 50 randomly selected images (Supplementary Table S1). The saturation range was split into two levels corresponding to unsaturated pixels [0.00, <0.50] and relatively saturated pixels [0.50, 1.00]. Consequently, each bin from the 14-bin histogram represents a pixel count of that particular color (14 features). The obtained color bins were normalized by the projected area of region of interest (ROI) presented in that image (ROI = root or ROI = shoot) resulting in percentages of color for each bin (14 features). For texture analysis, homogeneity, correlation, contrast, and entropy were retrieved using gray level co-occurrence matrix (four features). For geometric features, projected area, convex hull, minor and major axis length, perimeter, compactness, and solidity (seven features) were extracted from each plant. The area, length, and perimeter were converted to cm^2^ (three features). These features were extracted from roots and shoots separately for each plant.

During feature selection, only traits (derived from RGB images) that exhibited significant correlations with visual scores (*P* < 0.05) and were common across both datasets (GH_LSP, GH_RIL) were considered for further analysis. To reduce the effect of multicollinearity, the resulting subsets of features were used to build elastic net regression models using the “caret” package in R^1^. This penalized regression model is a combination of ridge regression and LASSO, which could be used as a feature selection method even with issues of large number of predictors and small number of samples (*n* < *p*) (Gonzales and De Saeger, 2018) or highly redundant information (Zou and Hastie, 2005). Data were normalized and centered to zero before using the model. Hyperparameters (α and λ) were optimized, each time, on the training data with 15-fold cross validation. Features were ranked by the importance of contribution in predicting visual scores, using several train and test dataset combinations. The initial model was developed using GH_LSP data as training set and GH_RIL data as testing set. Next, we manually evaluated the resulting features and removed the multicollinear features by keeping only the subset that led to the highest coefficient of determination (*R*^2^) and lowest root-mean-squared error on the testing set. Finally, the selected features were evaluated with four combinations of datasets: (1) GH_LSP data as training set and GH_RIL data as testing set; (2) 80% GH_LSP data as training set and 20% GH_LSP as testing set; (3) 80% GH_RIL data as training set and 20% GH_RIL as testing set; and (4) GH_RIL data as training set and GH_LSP data as testing set.

### Greenhouse Experiments: Hyperspectral Imaging, Image Processing, and Feature Extraction

Based on visual scores of ARR severity, 79 accessions from GH_LSP data and 21 lines from GH_RIL data were chosen from greenhouse experiments for hyperspectral imaging. These genotypes were taken to the laboratory for hyperspectral imaging immediately after collecting RGB images. A total of 2084 hypercubes or hyperspectral images (Table 1) were acquired using a push-broom type hyperspectral sensor (Hyperspec^^®^^ extended VNIR, Headwall Photonics Inc., Fitchburg, MA, United States) with 12 nm spectral resolution, 320 pixels spatial resolution, 145 spectral bands/channels (features), and 550–1700 nm wavelength range.

A UV–visible high-intensity quart tungsten halogen integrated with pulsed Xenon lamp (380–2500 nm, Headwall Photonics Inc., Fitchburg, MA, United States) was fixed on one side of hyperspectral camera (vertically mounted) at an angle of 45° to provide illumination for the camera’s field of view. Prior to imaging, distance from lens to sample and moving speed were set to 310 mm and 15 mm/s. Roots and shoots were scanned separately. Each resulting scan was a calibrated image (*I*_calib_), which is estimated using the following equation:

(1) $$I_{\text{calib}}=\frac{I_{\text{raw}}-I_{\text{dark}}}{I_{\text{white}}+I_{\text{dark}}}$$

where *I*_raw_ refers to the recorded raw hyperspectral image, *I*_dark_ refers to the dark reference image (∼0% reflectance) obtained without illumination unit and camera lens covered with its opaque black cap, and *I*_white_ refers to the white reference image obtained from a white reflectance panel (∼99% reflectance) with the illumination unit on. In GH_LSP, hyperspectral data were captured in the presence of indoor illumination; while in GH_RIL, the data were collected only in the presence of imaging system light source.

**FIGURE 3:**
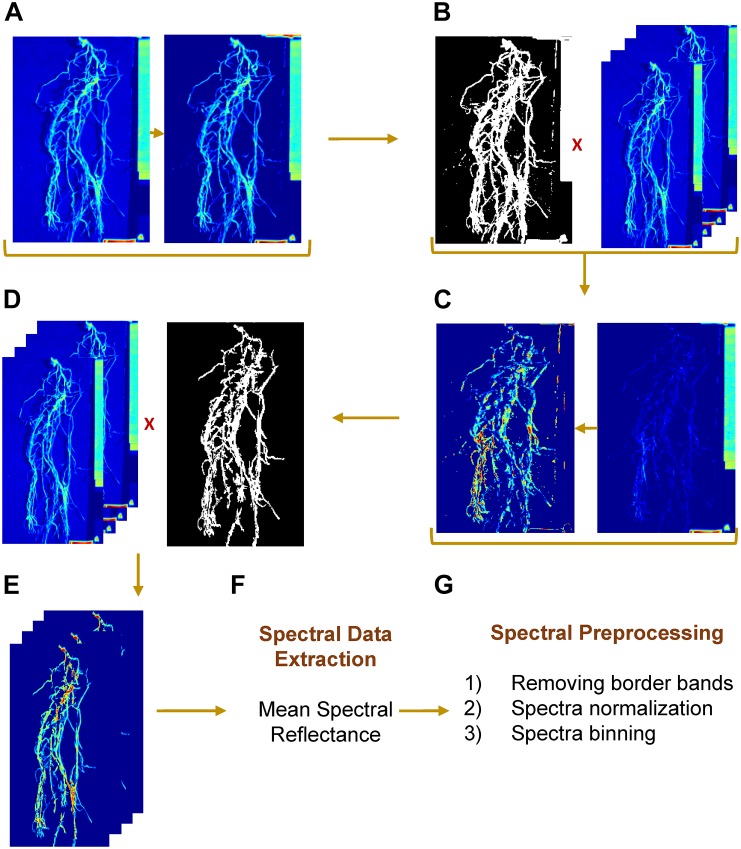
Hyperspectral root image processing and spectral preprocessing steps. **(A,B)** mask generation, **(C,D)** label removal, **(E)** resulting region of interest, **(F)** spectral data extraction, and **(G)** spectral preprocessing steps.

The hyperspectral data were acquired as band interleaved by line (BIL) format, which is a binary data stream. Each binary file was imported into MATLAB and converted to a 3-D matrix with 140 spectral bands. Before automating image processing, the spectral band “1038 nm” of the hypercube was selected to separate ROI – whether it is a shoot or root – from background (Figure 3A). The visual selection was based on the contrast between foreground and background. The resulting image was converted to a binary mask and was applied to the rest of channels (Figure 3B). Next, the spectral bands “1477” and “1535 nm” were selected, for shoot and root, respectively, to separate a white tag (that delimited the camera’s field of view) from ROI and to create a second mask by applying an intensity threshold (≥0.7 and ≥0.5 for shoot and root, respectively) and morphological operations by removing objects with total number of pixels lower than 130 (Figure 3C,D). The final image consisted of a gray scale image with a ROI as foreground and null pixels as background (Figure 3E). Mean spectral reflectance datum was extracted from each channel by averaging all foreground pixels (Figure 3F). Once the algorithm was developed and evaluated using a set of test images, the approach was implemented on all the images in both datasets (GH_LSP and GH_RIL).

Prior to the feature extraction, the initial and final ten spectral bands were removed to eliminate potential noisy signals. Each ROI (root/shoot) spectral signature was normalized such that the spectra had zero mean and unit variance. Then, the spectral bands/features were binned every 10 nm to represent multispectral band filters, resulting in a matrix of 99 spectral bands/features that was used in feature extraction (Figure 3G). In the first step of feature extraction, normalized difference spectral indices (NDSIs) were computed from hyperspectral data. NDSI is the normalized ratio of spectral reflectance of every combination of wavelengths, which were estimated using the following formula:

(2) $$\mathrm{NDSI}=\frac{{\mathrm{Band}}_{\text{k}}-{\mathrm{Band}}_{\text{n}}}{{\mathrm{Band}}_{\text{k}}+{\mathrm{Band}}_{\text{n}}}$$

where *Band*_k_ and *Band*_n_ represent reflectance at *k*th and *n*th spectral features (Inoue et al., 2008). With 99 spectral features and every combination of the spectral bands (permutation), the total number of features extracted were 9702. The feature selection methods were similar to the one described for RGB image feature extraction, where NDSI features were used, in place of RGB image features.

### Field Experiment: Multispectral Imaging, Image Processing, and Feature Extraction

For FE_RIL genotype evaluation, aerial images were collected using a quadcopter UAS (AgBot, ATI Inc., Oregon City, OR, United States). A five-band multispectral camera (RedEdge^TM^, Micasense Inc., Seattle, WA, United States), mounted on a gimbal of the UAS, was used to acquire the aerial data. The multispectral images acquired were red (668 ± 5 nm), green (560 ± 10 nm), blue (475 ± 10 nm), NIR (840 ± 20 nm), and red edge (717 ± 5 nm) bands with 1.2-megapixel resolution. Images were collected automatically according to a flight mission, in which the UAS was set to fly at 25 m above ground level and with a moving speed of 3 m/s allowing 80% horizontal and 70% vertical overlap. A reflectance panel (Spectralon Diffuse Reflectance Standard) used to correct the images was placed in the field during aerial data acquisition. Data acquisition was conducted on 44, 50, and 66 days after sowing (DAS). Field visual scores were taken on the same day or within 3 days of aerial data acquisition.

**FIGURE 4:**
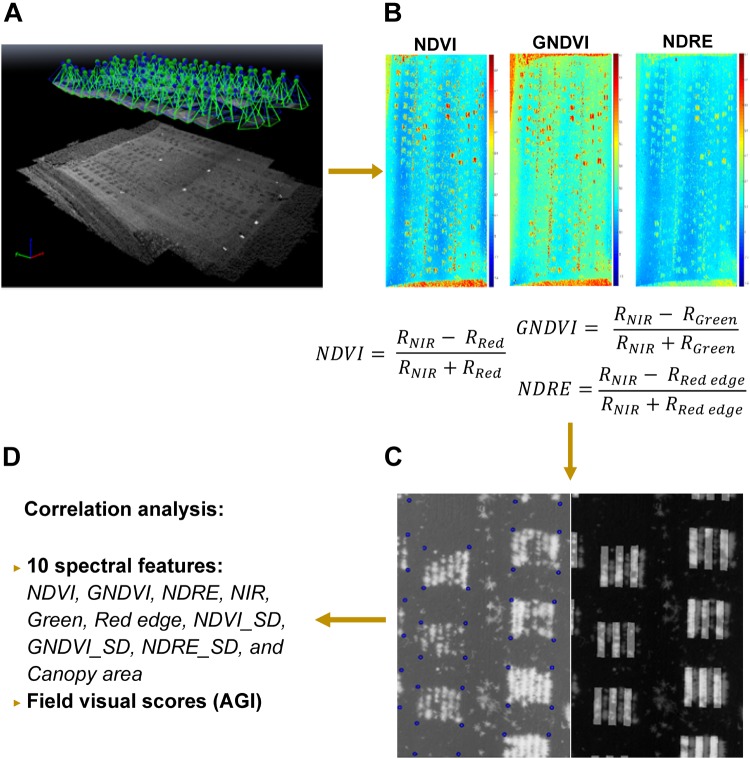
Procedure of multispectral image processing and data analysis: **(A)** image stitching in Pix4D; **(B)** index/reflectance maps generated in Pix4D; **(C)** plots identification and row separation; **(D)** statistical analysis of features. In **C**, corners of plots were highlighted by blue circles and masks for odd rows were overlapped with NDVI image for quality inspection.

The aerial images were first pre-processed in Pix4Dmapper (Pix4D Inc., San Francisco, CA, United States) to generate a nadir-view image that covered the entire field using the template for multispectral camera, shown in Figure 4A. After radiometric correction, six index/reflectance maps (normalized difference vegetation /NDVI, green NDVI/GNDVI, normalized difference red edge/NDRE, green, near-infrared/NIR, red edge) were created for further feature extraction (e.g., Figure 4B). Next, these maps were analyzed with a custom image processing algorithm developed in MATLAB to extract features from individual rows. Four corners of each plot were selected from the NDVI index map and their related positions were stored as coordinates (number of pixels from the origin of the image). The corners of plots were highlighted by blue circles right after being selected, and a sample image was shown in Figure 4C. In addition, a mask was generated by thresholding the NDVI index map using empirical thresholds (NDVI > 0, >0.22, and >0.25 for 44, 50, and 66 DAS, respectively). The resulting mask was used to separate the vegetation and soil in all the six index/reflectance maps. Using the coordinates and mask generated in the previous step, the algorithm separated six rows automatically in each plot, which is shown in Figure 4C, and extracted spectral features for lentil in each row. These features included NDVI, GNDVI, NDRE, NIR, green, red edge, NDVI_SD (standard deviation of NDVI within each row), GNDVI_SD, NDRE_SD, and canopy area. Finally, the extracted spectral features were exported as Excel file for statistical analysis (Figure 4D). The visual scores and image-derived features were also adjusted to account for soil variability following this formula:

(3) $${\mathrm{AdjScore}}_{\text{j}}={\mathrm{score}}_{\text{j}}-\left(\frac{{\mathrm{jscore}}_{01}+(N+1-j){\mathrm{score}}_{02}}{N+1}-\mathrm{m.score}\right)$$

where *AdjScore*_j_ is the adjusted disease score of the *j*th RIL plot located between two Avondale check plots, *score*_j_ is the non-adjusted disease score of the *j*th RIL plot, *score*_01_ and *score*_02_ are the disease scores of the two Avondale check plots located on both sides of the *j*th RIL plot, *m.score* is the mean disease score of all the Avondale check plots from the *i*th replicate, *N* is the total number of RIL plots between two Avondale check plots, and *j* is the value of the *j*th RIL plot (Dagnelie, 2003; Hamon et al., 2011). Correlation analysis was performed to investigate the relationship between features extracted from multispectral images and visual scores using both adjusted and non-adjusted data.

## Results

### Relationship Between Geometric Features and Plant Biomass

**FIGURE 5:**
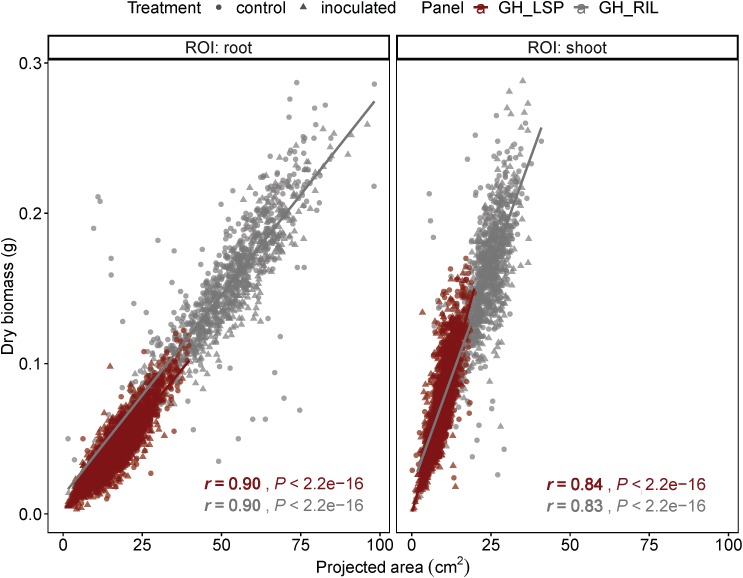
Correlation between projected area extracted from RGB images with root and shoot dry weight of plants from GH_LSP and GH_RIL lentil panels.

Geometric features extracted from RGB images, from both lentil panels (GH_LSP and GH_RIL), had significant correlations with manual measurements of biomass: root and shoot dry weights (0.10 *< r <* 0.90, *P <* 0.05). We found that correlation coefficients of projected area indicated a very good fit to the variation of manually measured dry weight (*r* = 0.90 for roots, *r* = 0.83–0.84 for shoots) (Figure 5). Due to the high variability in stem color, the image segmentation process resulted in removal of some pixels from shoot section, which could have reduced the level of correlation between digital shoot biomass and SDW. Nevertheless, the strong *r* value indicates that the digital biomass such as projected area is a good image-derived estimate of plant biomass and demonstrates the potential of RGB imaging as an accurate and high-throughput technique for measuring plant biomass.

### RGB-Derived Features Reflect Disease Symptoms

**FIGURE 6:**
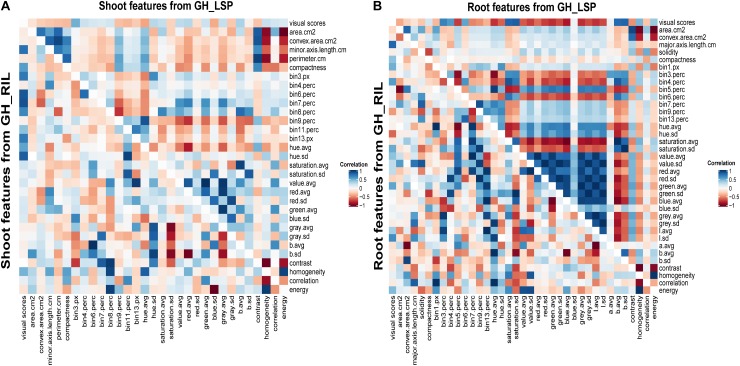
Heat maps of pairwise correlations of retained significant features of shoot and root sections extracted from two lentil panels: **(A)** shoot traits from GH_LSP genotypes (upper triangle) and shoot traits from GH_RIL genotypes (lower triangle); **(B)** root traits from GH_LSP genotypes (upper triangle) and root traits from GH_RIL genotypes (lower triangle). Note: diagonal data in **A** and **B** were changed to zero from one for better visualization and separation of GH_LSP and GH_RIL results. Supplementary Table S2 describes the image features.

Correlation analysis of RGB-derived phenotypic data obtained from the 351-lentil-single-line collection and 191-lentil-recombinant-inbred-line population revealed highly significant relationships with visual scores. In total, we identified 68 digital features (36 features extracted from root section and 32 features extracted from shoot section) common for both lentil panels that exhibited significant correlation with disease severity scores (features described in Supplementary Table S2). Interestingly, the correlation pattern between digital biomass features and visual scores was similar for both panels; where geometric descriptors extracted from root section expressed a negative relationship with disease severity (−0.38 < *r* < −0.07, *P* < 0.01) (Figure 6 and Supplementary Table S3). This observation indicates that susceptible plants – plants with high disease scores – could be associated with a decrease in digital biomass. However, this was only applicable for root dry biomass extracted from GH_LSP (*r* = −0.10, *P* < 0.05). Shoot dry biomass for the same panel was not significantly correlated with disease scores (*r* = −0.04). Whereas, both mean SDW and RDW extracted from GH_RIL image dataset were negatively and significantly correlated with mean visual scores (*r* = −0.12 and −0.16, *P* < 0.05). Furthermore, a significant relationship was observed between visual scores and color/texture features extracted from root section (0.06 < |*r*| < 0.83, *P* < 0.05) for both lentil panels. These relationships decreased for features extracted from shoot section, where geometric (−0.33 < *r* < −0.06, *P* < 0.05), color, and texture features (0.05 < |*r*| < 0.48, *P* < 0.05) had weaker but significant correlations with visual scores (Figure 6 and Supplementary Table S4).

**FIGURE 7:**
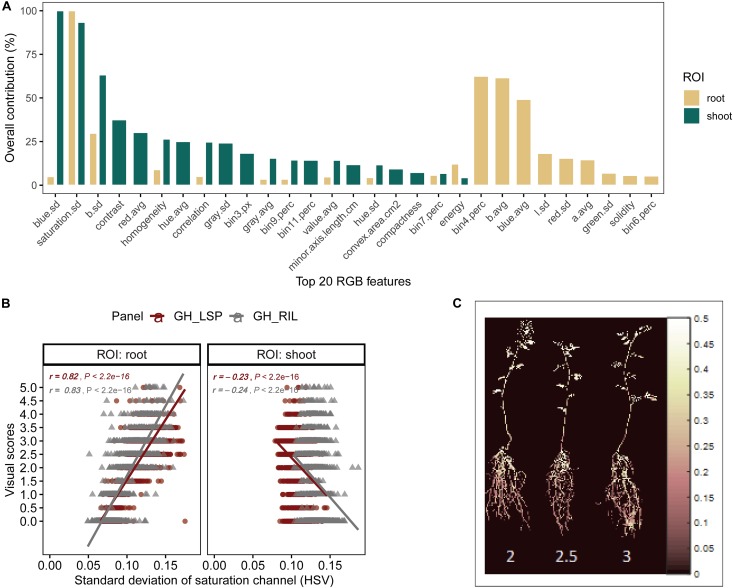
**(A)** Contribution of shoot and root features (top 20) to disease score prediction estimated by elastic net. **(B)** Comparison of Pearson’s correlation coefficients between the standard deviation of saturation channel (HSV) and visual scores across panels and region of interests. **(C)** Pseudocolor of saturation channel of inoculated lentils associated with disease scores (Avondale).

To validate the ability of the selected features – extracted from both root and shoot regions – to predict disease severity, we applied elastic net regression using GH_LSP data as a calibration set and GH_RIL data as testing set. In the shoot model, 29 common features (three features were eliminated by the model) were only able to explain 23% of the variability in disease scores with relatively low *R*^2^ (*R*^2^ = 0.23 and RMSE = 1.30) (e.g., Figure 7A). Whereas, in the root model, 27 common features (nine features were eliminated by the model) were able to significantly predict disease scores for GH_RIL data with higher *R*^2^ (*R*^2^ = 0.72 and RMSE = 1.25). Based on their contribution to the models, three final features were selected for root model (*R*^2^ = 0.67 and RMSE = 0.84), whereas, the shoot model did not show any improvement, even with removing lower ranked features (*p* = 11, *R*^2^ = 0.23, and RMSE = 1.34).

In addition to the validation of retained features across different panels, we tested their performances within each dataset (Table 2). For instance, models of image derived features from root section (*p* = 3) and shoot section (*p* = 11), that were trained and tested within GH_LSP data showed better prediction power (*R^2^* = 0.53 and 0.73 and RMSE = 0.88 and 0.66 for shoot and root, respectively) than models trained and tested within GH_RIL data (*R*^2^ = 0.32 and 0.67 and RMSE = 1.21 and 0.84 for shoot and root, respectively). However, it was found that models of root-derived features outperformed models of shoot-derived features in predicting visual scores and accounting for the most variance (around 70%), which aligns with the fact that symptoms of ARR are more visible in root section in the initial stages. However, both models of final root and shoot features exhibited similar prediction power of visual scores when the model was trained on GH_RIL and tested on GH_LSP (*R*^2^ = 0.42 and 0.45 and RMSE = 0.99 and 1.00 for shoot and root, respectively), which could be related to the high variability within RIL experiments.

**Table 2:** Prediction performances of final RGB features for shoot and root images within and across lentil panels.

Region of interest	Training set	Test set	*R*^2^	RMSE
	GH_LSP (100%)	GH_RIL (100%)	0.23	1.34
Shoot	GH_LSP (80%)	GH_LSP (20%)	0.53	0.88
(*p* = 11)	GH_RIL (80%)	GH_RIL (20%)	0.32	1.21
	GH_RIL (100%)	GH_LSP (100%)	0.42	0.99

	GH_LSP (100%)	GH_RIL (100%)	0.67	0.85
Root	GH_LSP (80%)	GH_LSP (20%)	0.73	0.66
(*p* = 3)	GH_RIL (80%)	GH_RIL (20%)	0.67	0.84
	GH_RIL (100%)	GH_LSP (100%)	0.45	1.00

**FIGURE 8:**
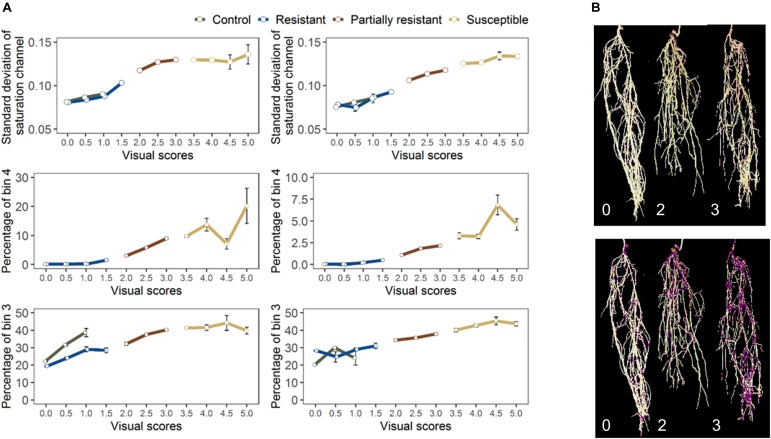
**(A)** Final retained features extracted from root section from GH_LSP genotypes (left) and GH_RIL genotypes (right) datasets. **(B)** Distribution of pixels from bin3 (bottom) and bin4 (top) in inoculated lentils (Avondale).

For the final retained features from root section, “saturation.sd” – standard deviation of saturation channel – (*r* = 0.82 and 0.83 for GH_LSP and GH_RIL, respectively), “bin3.perc” – percentage of bin3 – (*r* = 0.67 and 0.52), and “bin4.perc” – percentage of bin4 (*r* = 0.57 and 0.62) – were significantly and positively correlated with visual scores. Control and resistant plants from both lentil panels (GH_LSP and GH_RIL) had similar phenotypic pattern in almost all retained root features (Figure 8), except for percentage of bin3 extracted from HSV color space of control samples that were visually scored as “1” (Hue = [0.11, 0.15] and Saturation = [0, 0.5] and value = [0, 1]). In contrast, the susceptible group – samples visually scored between 3.5 and 5 – had higher values for these three features. To note, among the final retained features, only “saturation.sd” was common between root and shoot ROIs (Figure 7B,C). This feature revealed a different pattern for shoot region, where it was negatively correlated with disease scores (*r* = −0.23, *P* < 0.0001).

### Relationship Between Disease Severity Rating and Hyperspectral Spectra

**FIGURE 9:**
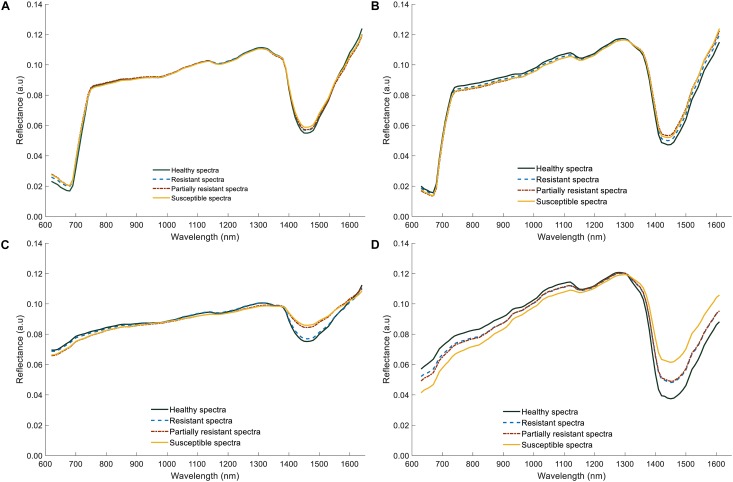
Average reflectance spectra of lentil (shoots and roots) for two lentil panels (GH_LSP and GH_RIL data). **(A)** Average spectral curves of lentil shoots in GH_LSP genotypes. **(B)** Average spectral curves of lentil shoots in GH_RIL genotypes. **(C)** Average spectral curves of lentil roots in GH_LSP genotypes. **(D)** Average spectral curves of lentil roots in GH_RIL genotypes.

The average reflectance spectra of shoot samples demonstrated typical green plant reflectance patterns, with minor spectral reflectance differences between the healthy and infected (Figure 9A,B). In contrast, significant differences could be discerned in the root spectra; infected roots had lower reflectance values in the visible region (600–780 nm, Figure 9C,D), than did healthy samples, probably resulting from darker appearance due to rotting. However, in the NIR region of 1300–1600 nm, reflectance values of both shoot and root infected samples were higher compared to healthy samples. Particularly, there was a larger difference in the reflectance values between healthy, resistant/partially resistant, and susceptible samples, near 1450 nm. Nonetheless, the average spectrum of roots did not capture any differences between resistant and partially resistant classes, which could indicate a similarity between these two groups.

**FIGURE 10:**
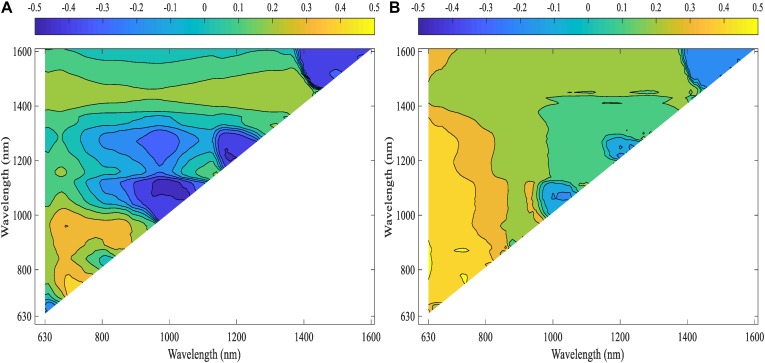
Correlation heat maps of computed NDSIs extracted from root section and visual scores for **(A)** GH_LSP data and **(B)** GH_RIL data.

Pearson’s correlation analysis was performed on the computed NDSIs of inoculated roots for both lentil panels (GH_LSP and GH_RIL) to investigate the relationship between root spectra and visual scores. We selected, at the first step, spectral features that were significantly correlated with disease scores and common for both datasets (Figure 10). Remarkably, we found that correlations were stronger for NDSIs extracted from GH_RIL population (0.15 < |*r*| < 0.50, *P* < 0.05) compared to GH_LSP population (0.11 < |*r*| < 0.45, *P* < 0.05). Here, not only the number of features were larger compared to number of samples but also hyperspectral data presented a problem of multicollinearity. In order to reduce data dimensionality, we trained elastic net regularized regression model with 15-fold cross validation on GH_LSP data and we validated the results using GH_RIL data. In this case, elastic net becomes more like ridge regression with α close to 1 to handle multicollinearity in the dataset. The model found, at this step, 1616 features with no significant contribution, where their coefficients were reduced to zero. The rest of features had explained around 30% of the variation in visual scores (*R*^2^ = 0.29 and RMSE = 1.74).

Although elastic net is very effective in removing correlated variables, the remaining features (number of features, *p* = 115) still were highly correlated with each other (*r* = 0.9–1.0). Therefore, we selected features with absolute value of coefficients greater than 2.5 (*p* = 35, *R*^2^ = 0.29 and RMSE = 1.77). Among the 35 features retained, 21 NDSIs were in the range of 630–830 nm and 14 in the range of 1320–1560 nm. Similarly, NDSIs in these two ranges were highly correlated to each other. Predictors in this case can provide the same information. Thus, we only retained, from the redundant NDSIs, predictors that were highly correlated with visual scores in both lentil panels. Such a reduction in number of features (*p* = 10) translated in a small reduction in *R*^2^ = 0.25 and RMSE = 1.64. Similar to the previous section, root model trained and tested with GH_LSP data showed higher prediction power (*R*^2^ = 0.54 and RMSE = 0.86) compared to the model trained and tested on GH_RIL data (*R*^2^ = 0.02 and RMSE = 1.5) (Table 3).

**Table 3:** Prediction performances of final NDSI features for shoot and root hyperspectral images within and across lentil panels.

Region of interest	Training set	Test set	*R*^2^	RMSE
	GH_LSP (100%)	GH_RIL (100%)	<0.01	3.09
Shoot	GH_LSP (80%)	GH_LSP (20%)	0.27	1.08
	GH_RIL (80%)	GH_RIL (20%)	0.01	1.38
	GH_RIL (100%)	GH_LSP (100%)	<0.01	1.57

	GH_LSP (100%)	GH_RIL (100%)	0.25	1.64
Root	GH_LSP (80%)	GH_LSP (20%)	0.54	0.86
	GH_RIL (80%)	GH_RIL (20%)	0.02	1.50
	GH_RIL (100%)	GH_LSP (100%)	0.15	3.34

However, for shoot models, only two NDSIs, in the range of 1160–1280 nm, were found significantly correlated with ARR visual scores and common across lentil panels (0.12 ≤ |*r*| ≤ 0.24, 0.05 < *P* < 0.0001), but showed no prediction power (whether trained on GH_LSP and tested on GH_RIL data or vice versa). The good performance of this model was exclusive only to GH_LSP (*R*^2^ = 0.27 and RMSE = 1.08) (Table 3). Root-derived NDSIs – computed from wavelengths of 700, 710, 730, and 790 nm – had strong and positive correlations with disease scores (0.35 ≤*r* ≤ 0.50, *P* < 0.0001). Whereas, in the range of 1350–1520 nm, the significance of correlation decreased for NDSIs extracted from GH_RIL (0.15 ≤ |*r*| ≤ 0.18, *P* < 0.05) compared to NDSIs extracted from GH_LSP (0.11 ≤ |*r*| ≤ 0.33, 0.05 < *P* < 0.0001) (Table 4). The small size of GH_RIL data could have contributed to these findings.

**Table 4:** Pearson’s correlation between retained NDSIs and visual scores for genotypes from two lentil panels (GH_LSP and GH_RIL).

			Coefficients of correlation	
Region of interest	Wavelength (nm) combination in NDSI		GH_LSP	GH_RIL
Shoot	1170	1160	0.24^∗∗∗^	−0.15^∗^
	1280	1270	−0.12^∗^	−0.15^∗^

Root	630	640	0.15^∗∗^	0.45^∗∗∗^
	660	650	0.19^∗∗^	−0.23^∗∗^
	670	660	0.11^∗^	−0.26^∗∗∗^
	1320	660	−0.12^∗^	−0.4^∗∗∗^
	700	710	0.45^∗∗∗^	0.43^∗∗∗^
	730	790	0.35^∗∗∗^	0.50^∗∗∗^
	840	790	−0.10^∗^	−0.45^∗∗∗^
	1350	1340	−0.11^∗^	−0.15^∗^
	1410	1420	0.29^∗∗∗^	0.17^∗^
	1530	1520	0.33^∗∗∗^	0.18^∗^

*Significance of correlation test: ^∗^P < 0.05; ^∗∗^P < 0.01; ^∗∗∗^P < 0.0001.*

### Field Phenotyping for Disease Symptoms

**FIGURE 11:**
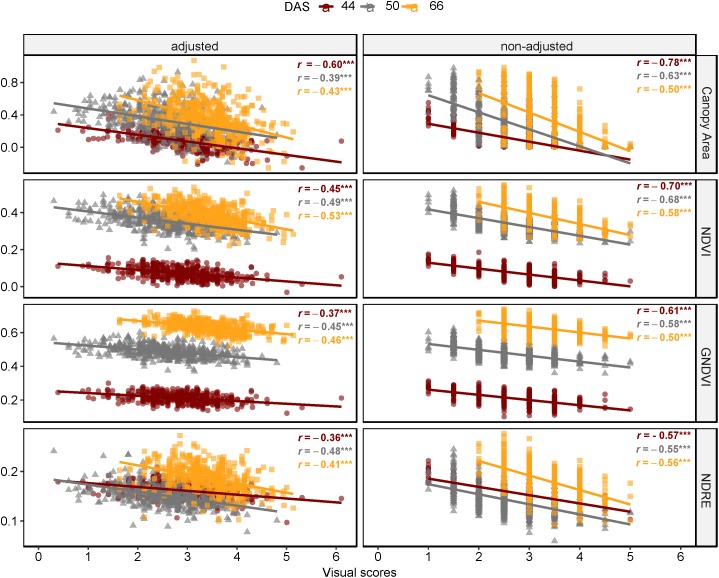
Comparison of Pearson’s coefficient of correlation across days after sowing and multispectral features as canopy area, NDVI, GNDVI, and NDRE (FE_RIL genotypes).

Results of correlation analysis of diseased lentil in the field experiment are shown in Supplementary Table S5 and Figure 11. Correlation coefficients between spectral features and field visual scores (AGI) varied across days after sowing and the type of features ranging from −0.78 to 0.51. Except for mean intensity of red edge band, all multispectral-derived features were significantly correlated with the AGI scores (*P* < 0.001). The correlation between non-adjusted spectral features and visual scores were generally higher compared to the adjusted data. Notably, the correlations between AGI and spectral indices and their derivatives (0.34 ≤ |*r*| ≤ 0.78, *P* < 0.0001) were also higher than those between AGI and individual bands (0.18 ≤*r* ≤ 0.44, *P* < 0.0001). The difference between *r* derived from non-adjusted and adjusted data reduced as the experiment advanced (from 44 to 66 DAS), which could also result from decline of correlation coefficients across adjusted and non-adjusted data.

## Discussion

Lentil is an important legume crop grown widely for its nutritional contribution to human diet and animal feed, and for its effects in improving soil fertility by fixing nitrogen (Infantino et al., 2006; Hamwieh et al., 2009; Foyer et al., 2016). However, the susceptibility of lentil to soil-borne pathogens such as *A. euteiches* could lead to severe losses in production (Gaulin et al., 2007). Therefore, plant breeding efforts are focusing on developing new lentil cultivars resistant to ARR (Ford et al., 1999; Infantino et al., 2006; Le May et al., 2017).

Disease tolerance is a complex trait to select for in plant breeding and crop improvement as it can be controlled by multiple genes (Nelson et al., 2018). Applicability of high-throughput plant phenotyping systems in deriving digital traits indicative of the health status in multiple plants subjected to biotic stresses is currently being investigated. In this study, we used high-throughput phenotyping methods as a faster, more accurate, and objective approach for screening ARR resistance in lentil. We evaluated 542 lentil genotypes (351 accessions collected worldwide and 191 RILs) using RGB imaging. Among these, 100 genotypes (79 LSP + 21 RIL) were further screened using hyperspectral imaging in greenhouse conditions and 173 RIL genotypes were evaluated in field conditions using multispectral aerial imaging at a flying altitude of 25 m.

Correlation analysis between retrieved digital traits and ground truth data, consisting of root and shoot dry biomass and ARR visual scores, were investigated and significant relationships were found. Indeed, most of the geometric/shape features extracted from RGB images including projected area, convex hull area, perimeter, compactness, solidity, major and minor axis lengths exhibited significant and positive correlations with dry weight (RDW and SDW). Notably, projected area showed the strongest correlation with dry weight, which can be considered as a good predictor for plant biomass. These findings were in line with a recent study by Chen et al. (2018), where a combination of relevant features – by incorporating physiological descriptors such as near-infrared intensity and geometric features such as projected area – were used to predict plant biomass. These additional features slightly enhanced the predictive power of models used compared to predicting plant biomass with only one single trait. In addition, we found that color and texture features derived from RGB images, NDSIs extracted from hyperspectral images, and multispectral indices from aerial images were significantly correlated with ARR disease scores.

Accordingly, application of high-throughput phenotyping platforms generates many features that cannot be evaluated manually or visually. Therefore, to predict ARR visual scores from extracted phenotypic traits, we built an elastic net penalized regression model. The choice for this model was based on the challenge of multicollinearity in our dataset, particularly in hyperspectral imaging data, in which, not only features were collinear, but also the total number of computed NDSIs was larger than the total number of samples from both years GH_LSP and GH_RIL data. Through this approach, we selected subsets of digital features extracted from RGB and hyperspectral images that resulted in accurately explaining the variance in disease scores. Intriguingly, in terms of model performances, features extracted from root section (three features from RGB images and 10 NDSIs from hyperspectral images) were able to accurately predict disease scores with coefficient of determination of (*R*^2^ = 0.67 and *R*^2^ = 0.25, respectively) when model were trained on GH_LSP and tested on GH_RIL data. These accuracies increased more when models were trained and tested on GH_LSP data but decreased when trained and tested on GH_RIL data. The differences in performance could be explained by the fact that features were selected at first from training the model with GH_LSP data and testing the model with GH_RIL data. Nevertheless, we confirmed the stability of RGB models trained on root features (0.45 ≤*R*^2^ ≤ 0.73), which could be explained by the fact that visual scores of ARR severity are closely associated with discoloration and browning of root tissues. For instance, saturation channel in the HSV color-space corresponds to the pureness or the depth of a certain color that is hard to perceive with human eyes (Sural et al., 2002). The ARR infection had resulted in necrosis and colored lesions on the root section (Chan and Close, 1987), which was translated by greater values of standard deviation of saturation channel. This feature indicates the dispersion of color-pureness and the range of variation in color shades, a reason that could explain why the mean of saturation channel failed to capture the spatial variability of pixel values in saturation channel. Additionally, in a recent study by Desgroux et al. (2018), a significant SNP marker was found associated with both resistance to ARR in peas and total root projected area as RSA trait. The projected root area was eliminated by elastic net models from initial steps because of it is high correlations with other features. Combining this trait with the final three selected RGB features slightly improved visual scores prediction results by a 2% (*R*^2^ = 0.68 and RMSE = 0.83) when GH_LSP was used as training set and GH_RIL as testing set. On the other hand, the moderate performance of models developed from hyperspectral features could be related to the fact that ARR resistance is a complex trait and descriptors from visible range, visual scores, or RGB traits, are not enough to decipher the disease interaction with lentil roots in near-infrared region.

The phenotypic response patterns to *A. euteiches* infection differed between root and shoot. For instance, we found, from multispectral traits extracted from FE_RIL experiment, that percentage of canopy area, NDVI, GNDVI, and NDRE were negatively correlated (Figure 11) with AGI. This finding could indicate that ARR resistance is associated with high canopy cover. In contrast to foliar diseases, several studies explained that a high foliage cover of the above ground or “aerial organs” will create a favorable humid microclimate for disease development (Calonnec et al., 2013; Richard et al., 2013; Desgroux et al., 2018). As for roots, projected area along with other geometric RGB traits were negatively correlated with visual scores, which supports previous findings (Djébali et al., 2009; Desgroux et al., 2018). Furthermore, the decreasing pattern of multispectral traits associated with higher AGI could be explained by the senescence/necrosis of foliage. For instance, several studies have found low NDVI/GNDVI values are associated with stressed plants and low chlorophyll concentrations (Richardson et al., 2002).

Hyperspectral imaging provided a high-dimensional reflectance data with information expanding over 140 spectral bands, with more than 110 spectral bands in the near-infrared region. Although HSI imaging demonstrated a great potential for phenotyping disease resistance in literature, in our study and with our adopted approach, this imaging technique showed a moderate performance. Such performance could have resulted from the type of validation data as the ground-truth relied only on disease visual scores. This technique could be improved by extracting other types of ground-truth data (e.g., biochemical constituents, gene expression). A similar finding was observed with multispectral imaging. With two spectral bands in near-infrared region, vegetation indices such as NDVI, GNDVI, and NDRE were significantly and moderately correlated with disease scores in field conditions. Although root RGB-derived features were highly correlated with disease scores in comparison to shoot RGB-derived features in controlled environment, the same may not be practical in field conditions, given that only above ground lentils/legumes biomass are assessed. Considering the need for rapid screening for above ground lentils/legumes in field conditions, UAV coupled with multispectral imaging could enable a faster assessment for ARR severity. The selection of one of these imaging techniques relies on three main questions: (1) how big is the variability of disease expression within lines/accessions? (to select the appropriate image resolution), (2) what is the type of ground-truth data? (to validate the acquired image features), and (3) is the assessment in controlled environment or field conditions? (to decide on the feasibility of imaging). It should be noted that although results from all imaging techniques were promising, the reliability of these extracted features can be further strengthened on integration with genetic studies.

In this study, we identified RGB, multispectral, and hyperspectral traits that exhibited significant correlations with visual scores. The validation of such high-throughput methods, however, depends largely on the quality of image acquisition (Lobet, 2017). The more standardized the protocol of imaging is, the more automatic and faster the data processing will be. The results from our approach were highly influenced by the variation of disease symptoms among lentil plants within accessions/lines, even among the checks. This variation was translated by a variability in disease scores even for cultivar checks, which could be addressed by taking the average score for each line or accession. However, we preferred to investigate the efficiency of our approach at an individual plant level in order to correspond with the plant breeder’s rating. As a further step toward adopting such approaches in evaluating *A. euteiches* severity in lentil, digital traits will be integrated in QTL analysis to check for related expressions.

## Author Contributions

YM contributed to inoculation and plantation. AM, YM, and CZ contributed to data collection. AM and SS contributed to data processing and analysis – RGB and hyperspectral data, writing, and original draft preparation. CZ and SS contributed to data processing and analysis – UAV images. SS, RM, CC, and DM contributed to resources and funding acquisition. CZ, YM, RM, and CC contributed to writing, review, and editing. SS and RM contributed to supervision and project administration.

## Conflict of Interest Statement

The authors declare that the research was conducted in the absence of any commercial or financial relationships that could be construed as a potential conflict of interest. The handling Editor declared a shared affiliation, though no other collaboration with several of the authors RM and CC at the time of review.

## Footnotes

1 http://www.r-project.org/; release 3.5.1
