# Supplementary material for: Advanced Imaging for Quantitative Evaluation of Aphanomyces Root Rot Resistance in Lentil
Source: Front Plant Sci. 2019 Apr 16;10:383. doi: 10.3389/fpls.2019.00383 (PMC6477098; doi:10.3389/fpls.2019.00383)
Supplement: Supplementary file 1 [file Data_Sheet_1.docx]

Supplementary Material

Advanced Imaging for Quantitative Evaluation of Aphanomyces Root Rot Resistance in Lentil

Afef Marzougui^1^, Yu Ma^2^, Chongyuan Zhang^1^, Rebecca J. McGee^2^, Clarice J. Coyne^3^, Dorrie Main^4^, Sindhuja Sankaran^1^*

***Correspondance:** Dr. Sindhuja Sankaran: sindhuja.sankaran@wsu.edu

# Supplementary Figures and Tables

## Supplementary Figures


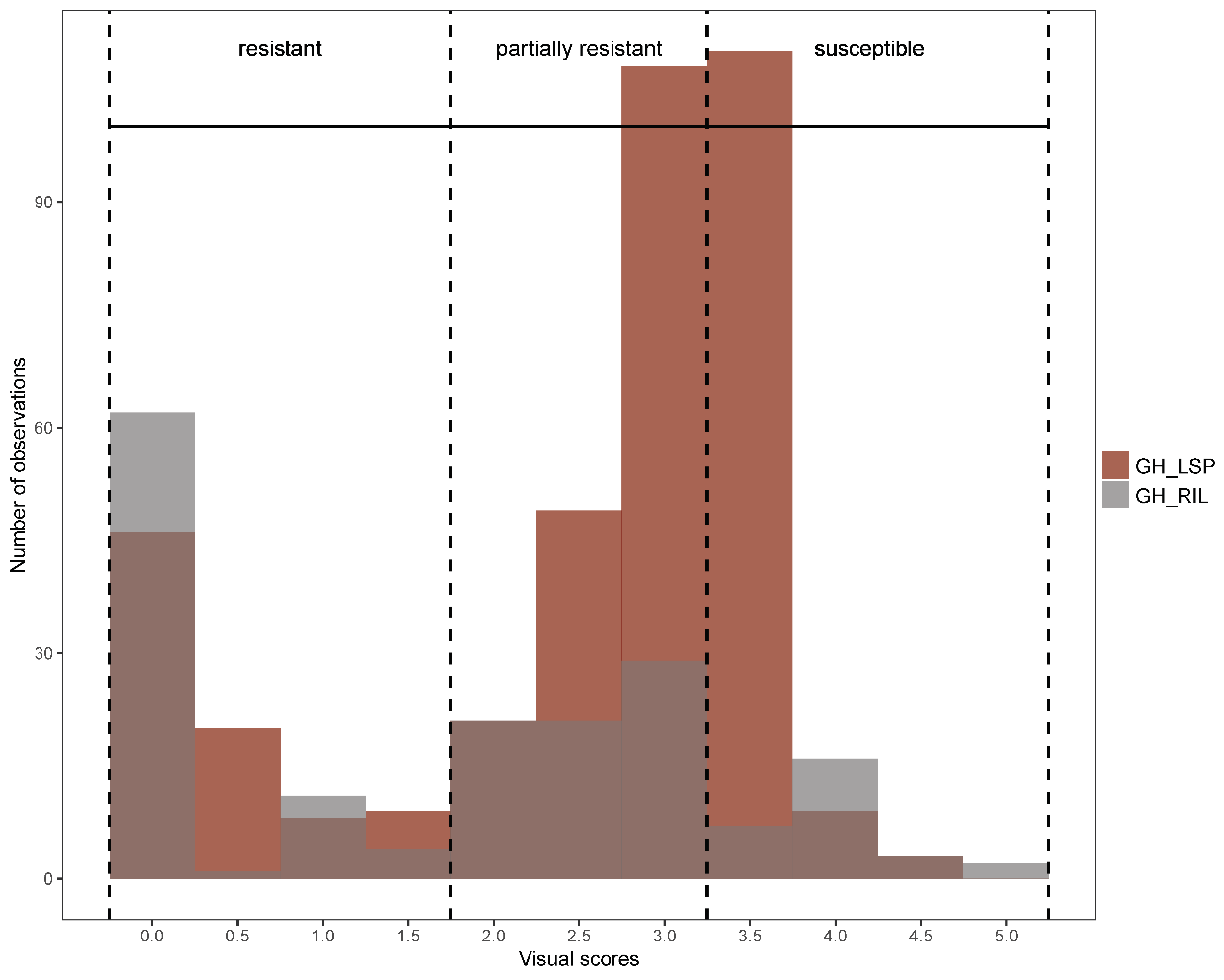


**Supplementary Figure S1.** Distribution of disease visual scores across two lentil panels.


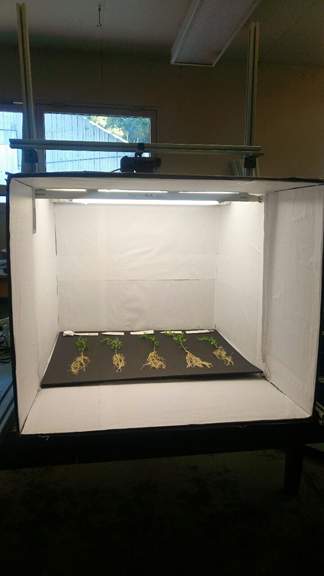


Supplementary Figure S2. Phenotyping box used for Red-Green-Blue imaging.

## Supplementary Tables

| **Bin number** | **Interval range** |
| --- | --- |
| 1 | 0.05 ≤ Hue ≤ 0.10 and 0.00 ≤ Saturation < 0.50 and 0.00 ≤ Value ≤ 1.00 |
| 2 | 0.05 ≤ Hue ≤ 0.10 and 0.50 ≤ Saturation ≤ 1.00 and 0.00 ≤ Value ≤ 1.00 |
| 3 | 0.11 ≤ Hue ≤ 0.15 and 0.00 ≤ Saturation < 0.50 and 0.00 ≤ Value ≤ 1.00 |
| 4 | 0.11 ≤ Hue ≤ 0.15 and 0.50 ≤ Saturation ≤ 1.00 and 0.00 ≤ Value ≤ 1.00 |
| 5 | 0.151 ≤ Hue ≤ 0.20 and 0.00 ≤ Saturation < 0.50 and 0.00 ≤ Value ≤ 1.00 |
| 6 | 0.151 ≤ Hue ≤ 0.20 and 0.50 ≤ Saturation ≤ 1.00 and 0.00 ≤ Value ≤ 1.00 |
| 7 | 0.21 ≤ Hue ≤ 0.25 and 0.00 ≤ Saturation < 0.50 and 0.00 ≤ Value ≤ 1.00 |
| 8 | 0.21 ≤ Hue ≤ 0.25 and 0.50 ≤ Saturation ≤ 1.00 and 0.00 ≤ Value ≤ 1.00 |
| 9 | 0.251 ≤ Hue ≤ 0.30 and 0.00 ≤ Saturation < 0.50 and 0.00 ≤ Value ≤ 1.00 |
| 10 | 0.251 ≤ Hue ≤ 0.30 and 0.50 ≤ Saturation ≤ 1.00 and 0.00 ≤ Value ≤ 1.00 |
| 11 | 0.31 ≤ Hue ≤ 0.35 and 0.00 ≤ Saturation < 0.50 and 0.00 ≤ Value ≤ 1.00 |
| 12 | 0.31 ≤ Hue ≤ 0.35 and 0.50 ≤ Saturation ≤ 1.00 and 0.00 ≤ Value ≤ 1.00 |
| 13 | 0.351 ≤ Hue ≤ 0.40 and 0.00 ≤ Saturation < 0.50 and 0.00 ≤ Value ≤ 1.00 |
| 14 | 0.351 ≤ Hue ≤ 0.40 and 0.50 ≤ Saturation ≤ 1.00 and 0.00 ≤ Value ≤ 1.00 |

Supplementary Table S1. Interval ranges of bins generated from HSV color histogram

Supplementary Table S2. Overview for common traits extracted from shoot and root samples.

| **Trait ID** | **Definition** |
| --- | --- |
| area.cm2 | Projected area/total number of pixels converted to cm^2^ |
| convex.area.cm2 | Total area of the convex hull in cm^2^ |
| minor.axis.length.cm | Length of minor axis in cm |
| major.axis.length.cm | Length of major axis in cm |
| perimeter.cm | Total distance of object (root/shoot) boundary in cm |
| solidity | The ratio of projected area and convex hull |
| compactness | The ratio of projected area and perimeter |
| bin1.px | Total number of pixels in bin number 1 (refer to Table S1) |
| bin3.perc | Percentage of pixels in bin number 3 (refer to Table S1) |
| bin3.px | Total number of pixels in bin number 3 (refer to Table S1) |
| bin4.perc | Percentage of pixels in bin number 4 (refer to Table S1) |
| bin5.perc | Percentage of pixels in bin number 5 (refer to Table S1) |
| bin6.perc | Percentage of pixels in bin number 6 (refer to Table S1) |
| bin7.perc | Percentage of pixels in bin number 7 (refer to Table S1) |
| bin8.perc | Percentage of pixels in bin number 8 (refer to Table S1) |
| bin9.perc | Percentage of pixels in bin number 9 (refer to Table S1) |
| bin11.perc | Percentage of pixels in bin number 11 (refer to Table S1) |
| bin13.px | Total number of pixels in bin number 13 (refer to Table S1) |
| bin13.perc | Percentage of pixels in bin number 13 (refer to Table S1) |
| hue.avg | Average intensity of hue channel |
| hue.sd | Standard deviation of hue channel |
| saturation.avg | Average intensity of saturation channel |
| saturation.sd | Standard deviation of saturation channel |
| value.avg | Average intensity of value channel |
| value.sd | Standard deviation of value channel |
| red.avg | Average intensity of red channel |
| red.sd | Standard deviation of red channel |
| green.avg | Average intensity of green channel |
| green.sd | Standard deviation of green channel |
| blue.avg | Average intensity of blue channel |
| blue.sd | Standard deviation of blue channel |
| gray.avg | Average intensity of gray image |
| gray.sd | Standard deviation of gray image |
| l.avg | Average intensity of L channel |
| l.sd | Standard deviation of L channel |
| a.avg | Average intensity of a channel |
| b.avg | Average intensity of b channel |
| b.sd | Standard deviation of b channel |
| contrast | Intensity contrast |
| homogeneity | Distribution of elements in Gray-Level Co-occurrence Matrix (GLCM) |

Supplementary Table S3. Pearson’s correlation coefficient between common root features extracted from inoculated samples and visual scores. Highlighted traits correspond to the final selected features used in elastic net model. Significance of correlation test: *P<0.05; **P<0.01; ***P<0.0001

|  | Pearson’s coefficient of correlation | |
| --- | --- | --- |
|  | GH_LSP  (n = 1717) | GH_RIL  (n = 1616) |
| area.cm2 | -0.38*** | -0.20*** |
| convex.area.cm2 | -0.24*** | -0.11*** |
| major.axis.length.cm | -0.14*** | -0.07** |
| solidity | -0.27*** | -0.16*** |
| compactness | -0.20*** | 0.14*** |
| bin1.px | -0.33*** | -0.12*** |
| **bin3.perc** | 0.67*** | 0.52*** |
| **bin4.perc** | 0.57*** | 0.62*** |
| bin5.perc | -0.70*** | -0.55*** |
| bin6.perc | 0.53*** | 0.54*** |
| bin7.perc | -0.49*** | -0.19*** |
| bin9.perc | -0.45*** | -0.15*** |
| bin13.perc | -0.35*** | -0.13*** |
| hue.avg | -0.73*** | -0.50*** |
| hue.sd | -0.60*** | -0.35*** |
| saturation.avg | 0.80*** | 0.74*** |
| **saturation.sd** | 0.82*** | 0.83*** |
| value.avg | -0.62*** | -0.50*** |
| value.sd | -0.69*** | -0.33*** |
| red.avg | -0.56*** | -0.45*** |
| red.sd | -0.69*** | -0.33*** |
| green.avg | -0.74*** | -0.64*** |
| green.sd | -0.66*** | -0.10*** |
| blue.avg | -0.81*** | -0.72*** |
| blue.sd | 0.06* | 0.66*** |
| gray.avg | -0.74*** | -0.63*** |
| gray.sd | -0.66*** | -0.12*** |
| l.avg | -0.73*** | -0.62*** |
| l.sd | -0.66*** | -0.11*** |
| a.avg | 0.06* | 0.05* |
| b.avg | 0.78*** | 0.69*** |
| b.sd | 0.80*** | 0.81*** |
| contrast | -0.46*** | -0.18*** |
| homogeneity | 0.46*** | 0.18*** |
| correlation | -0.28*** | -0.33*** |
| energy | 0.38*** | 0.19*** |

Supplementary Table S4. Pearson’s correlation coefficient between common shoot features extracted from inoculated samples and visual scores. Highlighted traits correspond to the final selected features used in elastic net model. Significance of correlation test: *P<0.05; **P<0.01; ***P<0.0001

|  | Pearson’s coefficient of correlation | |
| --- | --- | --- |
|  | GH_LSP  (n = 1717) | GH_RIL  (n = 1616) |
| area.cm2 | -0.15*** | -0.09*** |
| convex.area.cm2 | -0.25*** | -0.07** |
| minor.axis.length.cm | -0.28*** | -0.12*** |
| perimeter.cm | -0.30*** | -0.09** |
| compactness | -0.33*** | -0.06* |
| **bin3.px** | 0.48*** | 0.20*** |
| bin4.perc | 0.22*** | 0.14*** |
| bin6.perc | 0.17*** | 0.08*** |
| bin7.perc | 0.15*** | 0.21*** |
| bin8.perc | 0.19*** | 0.19*** |
| bin9.perc | -0.31*** | -0.18*** |
| bin11.perc | -0.34*** | -0.11*** |
| bin13.px | -0.26*** | -0.13*** |
| **hue.avg** | -0.40*** | -0.18*** |
| hue.sd | 0.28*** | 0.07*** |
| saturation.avg | 0.24*** | 0.12*** |
| **saturation.sd** | -0.23*** | -0.24*** |
| value.avg | 0.26*** | 0.15*** |
| **red.avg** | 0.28*** | 0.16*** |
| red.sd | -0.18*** | -0.14*** |
| green.avg | 0.22*** | 0.12*** |
| **blue.sd** | -0.38*** | -0.30*** |
| **gray.avg** | 0.23*** | 0.13*** |
| gray.sd | -0.27*** | -0.16*** |
| l.avg | 0.24*** | 0.13*** |
| l.sd | -0.23*** | -0.12*** |
| b.avg | 0.32*** | 0.18*** |
| **b.sd** | -0.05* | -0.10** |
| **contrast** | -0.29*** | -0.11*** |
| homogeneity | 0.23*** | 0.08** |
| **correlation** | 0.19*** | 0.05* |
| energy | 0.16*** | 0.09*** |

Supplementary Table S5. Comparison of Pearson’s correlation across multispectral features extracted from remote sensing data and days after sowing (DAS). Significance of correlation test: *P<0.05; **P<0.01; ***P<0.0001

| DAS | Adjustment | Canopy area | NDVI | NDVI_SD | GNDVI | GNDVI_SD | NDRE | NDRE_SD | NIR | Green | Red edge |
| --- | --- | --- | --- | --- | --- | --- | --- | --- | --- | --- | --- |
| 44 | Non-Adj | -0.78*** | -0.70*** | -0.69*** | -0.61*** | -0.67*** | -0.57*** | -0.64*** | 0.44*** | 0.37*** | 0.05 |
|  | Adjusted | -0.60*** | -0.45*** | -0.48*** | -0.37*** | -0.46*** | -0.36*** | -0.37*** | 0.22*** | 0.23*** | 0.01 |
| 50 | Non-Adj | -0.63*** | -0.68*** | -0.65*** | -0.58*** | -0.63*** | -0.55*** | -0.52*** | 0.21*** | 0.51*** | 0.21*** |
|  | Adjusted | -0.39*** | -0.49*** | -0.53*** | -0.45*** | -0.50*** | -0.48*** | -0.44*** | 0.24*** | 0.33*** | 0.04 |
| 66 | Non-Adj | -0.50*** | -0.58*** | -0.49*** | -0.50*** | -0.46*** | -0.56*** | -0.34*** | 0.24*** | 0.43*** | 0.18*** |
|  | Adjusted | -0.43*** | -0.53*** | -0.47*** | -0.46*** | -0.47*** | -0.41*** | -0.38*** | 0.20*** | 0.36*** | 0.05 |
